# Plasma Cathepsin S and Cathepsin S/Cystatin C Ratios Are Potential Biomarkers for COPD

**DOI:** 10.1155/2016/4093870

**Published:** 2016-11-22

**Authors:** Takahiro Nakajima, Hidetoshi Nakamura, Caroline A. Owen, Shuichi Yoshida, Keishi Tsuduki, Shotaro Chubachi, Toru Shirahata, Shuko Mashimo, Miho Nakamura, Saeko Takahashi, Naoto Minematsu, Hiroki Tateno, Seitaro Fujishima, Koichiro Asano, Bartolome R. Celli, Tomoko Betsuyaku

**Affiliations:** ^1^Division of Pulmonary Medicine, Department of Medicine, Keio University School of Medicine, 35 Shinanomachi, Shinjuku-ku, Tokyo 160-8582, Japan; ^2^Department of Respiratory Medicine, Saitama Medical University, 38 Morohongo, Moroyama-machi, Iruma-gun, Saitama 350-0495, Japan; ^3^Division of Pulmonary and Critical Care Medicine, Brigham and Women's Hospital and Harvard Medical School, 75 Francis Street, Boston, MA 02115, USA; ^4^Center for General Internal Medicine and Education, Keio University School of Medicine, 35 Shinanomachi, Shinjuku-ku, Tokyo 160-8582, Japan; ^5^Division of Pulmonary Medicine, Department of Medicine, Tokai University School of Medicine, 143 Shimokasuya, Isehara-shi, Kanagawa 259-1193, Japan

## Abstract

*Purpose*. This study aimed to examine whether plasma levels of cathepsin S or its inhibitor, cystatin C, may serve as biomarkers for COPD.* Patients and Methods*. We measured anthropometrics and performed pulmonary function tests and chest CT scans on 94 patients with COPD and 31 subjects with productive cough but no airflow obstruction (“at risk”; AR). In these subjects and in 52 healthy nonsmokers (NS) and 66 healthy smokers (HS) we measured plasma concentrations of cathepsin S and cystatin C using an ELISA. Data were analyzed using simple and logistic regression and receiver operating characteristic analyses.* Results*. Cathepsin S and cystatin C plasma levels were significantly higher in the COPD and AR groups than in the NS and HS groups (*p* < 0.01). Among the COPD patients and AR subjects, plasma cathepsin S levels and cathepsin S/cystatin C ratios, but not cystatin C levels, were negatively related to severe airflow limitation (% FEV_1_ predicted < 50%; *p* = 0.005) and severe emphysema as assessed by low attenuation area (LAA) score on chest CT scans (LAA ≥ 8.0; *p* = 0.001).* Conclusion*. Plasma cathepsin S and cathepsin S/cystatin C ratios may serve as potential biomarkers for COPD.

## 1. Introduction

COPD is characterized by narrowing of small airways and lung parenchymal destruction, partly caused by chronic lung inflammation including increased numbers of neutrophils, macrophages, and CD8-positive T lymphocytes, oxidative stress, and alveolar septal cell apoptosis [1–3]. Changes in the % FEV_1_ predicted and clinical parameters such as exacerbation rates are currently the most commonly used endpoints for monitoring responses to therapies in COPD patients, but these measures have limitations including a relatively long duration of treatment and large number of subjects needed for study in order to detect the changes in response to therapy. Consequently, there is an urgent need to identify and validate sensitive and reliable biomarkers for COPD in compartments that are easy to sample, such as blood, to more quickly assess the efficacy of novel therapies for COPD in clinical trials. In particular, it will be crucially important to identify reliable biomarkers for early stage COPD, the stage at which novel therapies might have the most impact on successfully modifying the course of the disease and preserving lung function.

Recent research has identified a number of potential molecules and pathways that could serve as potential biomarkers and be targeted to develop novel therapies [4]. One such group of molecules is proteinases. There is compelling evidence that an imbalance between the levels of proteinases and their inhibitors in the lung contributes to emphysema development. Proteinases such as neutrophil elastase, matrix metalloproteases, and cysteine proteases contribute to emphysema development and/or small airway remodeling in COPD [5]. Another proteinase that has been implicated in COPD pathogenesis is cathepsin S, a lysosomal acidic proteinase which is highly expressed by cells implicated in COPD pathogenesis including macrophages and dendritic cells [6]. Cathepsin S level and activity in bronchoalveolar lavage fluid from COPD patients are higher than those from healthy volunteers [7]. Additionally, cathepsin S is a potent elastin-degrading proteinase and participates in the adaptive immune response [8]. Studies of murine models of emphysema have implicated cathepsin S in promoting lung inflammation and destruction [9]. The major inhibitor of cathepsin S in extracellular fluids including plasma is cystatin C which is expressed by alveolar macrophages, bronchioepithelial cells, and endothelial cells [10–12]. However, this inhibitor has not been well studied in the context of COPD and neither cathepsin S nor cystatin C levels have been measured in plasma samples from human COPD patients.

We hypothesized that plasma concentrations of cathepsin S and cystatin C would differ between COPD patients, subjects with cough and sputum, and healthy smokers and nonsmokers. To test this hypothesis, we measured the plasma concentrations of cathepsin S and cystatin C and explored the association between levels of these biomarkers with degree of airflow obstruction and emphysema severity as assessed by low attenuation areas on chest CT scans and diffusing capacity of the lung for carbon monoxide (DL_CO_) measurements.

## 2. Materials and Methods

### 2.1. Study Populations

This study subjects included 125 patients suggestive of COPD consecutively visiting the outpatient clinic of Keio University Hospital from 2000 through 2003 and also included 118 healthy volunteers (age ≥ 50 years). All patients underwent pulmonary function tests (PFT) and chest CT. The patients suspected of having clinically stable COPD fulfilled the following criteria: (1) smoking history of ≥ 10 pack-years or more; (2) age ≥ 40 years old; and (3) having chronic respiratory symptoms including cough, sputum, and dyspnea on exertion. The patients were excluded if they were (1) having any pulmonary disease other than COPD; (2) having serum creatinine level > 1.5 mg/dL with renal dysfunction; (3) having serious comorbidities including unstable cardiovascular or cerebral diseases and treated malignant tumors; (4) having self-reported asthma; and (5) having an exacerbation for at least one month prior to recruitment or being treated with oral corticosteroids. The COPD patients (*n* = 94) were diagnosed based on airflow obstruction as defined by a ratio of forced expiratory volume in 1 second to forced vital capacity (FEV_1_/FVC) lower than 0.7 and an % FEV_1_ predicted value lower than the 95% confidence interval for this population by PFT. One group of 31 subjects (at risk of developing COPD; AR) was defined as having chronic respiratory symptoms (cough, sputum, and dyspnea on exertion) but no airflow limitation as assessed by PFT. Enrolled healthy volunteers were classified into two groups by smoking history: 52 healthy nonsmokers (NS) and 66 healthy smokers (HS). This study was approved by the Ethics Committee of Keio University Hospital and informed consent was obtained from each subject.

### 2.2. Measurement of Concentrations of Cathepsin S and Cystatin C

Plasma concentrations of total cathepsin S were measured in duplicate using the enzyme-linked immunosorbent assay (ELISA) kit (R&D systems, Inc. Minneapolis, MN, USA) after diluting the samples 1 : 100 using the diluent supplied by the kit manufacturer. The lower detectable limit of cathepsin S using these kits was 15.6 pg/mL. Plasma concentrations of cystatin C were determined in duplicate using a sandwich ELISA as previously described [13]. GT 13 was used as a mouse anti-cystatin C monoclonal antibody. Plasma was also 100 times diluted with phosphate buffered saline before the assay. The lower detectable limit of cystatin C using this assay was 1.9 ng/mL.

### 2.3. Assessment of Clinical Parameters

Spirometry was performed in all patients of the COPD and AR groups using an electronic spirometer (MFR-8200; Nihon Kohden, Tokyo, Japan). Regular treatment was not changed prior to spirometric testing. DL_CO_ was estimated by 10 s breath holding in most patients of the COPD (*n* = 83) and AR (*n* = 20) groups (*n* = 103) (Chestac-55V; Chest, Tokyo, Japan). Chest CT was also performed in all patients of the COPD and AR groups (Proseed, GE; Yokogawa Medical Systems, Tokyo, Japan).

To calculate emphysema severity, the entire lung was divided into six zones (i.e., left and right zones in the upper, middle, and lower lung fields). Low attenuation areas (LAA) were visually scored in each zone on a scale from 0 to 4: 0, no LAA; 1, 1–25%; 2, 26–50%; 3, 51–75%; 4, 76–100%. The total (0–24) was assessed by three pulmonologists in a blinded manner and the mean score was defined as the LAA score, a quantitative indicator of emphysematous change [14, 15]. Diameters of main pulmonary artery and abdominal aorta at the celiac artery were also measured on the chest CT images [16].

### 2.4. Statistical Analysis

Data were expressed as the mean ± SD. Plasma cathepsin S and cystatin C levels were compared among the groups by analysis of variance and the Scheffé test. Correlations between the plasma concentrations of cathepsin S and cystatin C and clinical parameters were examined using simple linear regression analysis. Independent associations between the plasma concentrations of cathepsin S and cystatin C and severe airflow limitation (% FEV_1_ predicted < 50%) or emphysematous change (LAA ≥ 8.0) were determined using logistic regression analysis. Logistic regression analysis models were prepared to avoid the influence of potential confounders, including age, sex, body mass index (BMI), smoking status, serum creatinine levels, the presence of hypertension, and treatment with inhaled corticosteroid (ICS). Inclusion of variables was based on existing knowledge of risk factors for COPD and on the factors related to plasma cystatin C levels according to previous reports [17, 18]. We followed standard method to estimate sample size for logistic regression analysis, with at least ten outcomes needed for each included independent variable. Receiver operating characteristic (ROC) analyses of plasma cathepsin S and cystatin C levels and the cathepsin S/cystatin C ratio were performed to examine the sensitivity and specificity of plasma cathepsin S and cystatin C levels as biomarkers for COPD in all participants. *p* values < 0.05 were considered statistically significant.

## 3. Results

### 3.1. Characteristics of Study Populations

As shown in Table 1, the mean age in the COPD group was older than the other three groups. The COPD group consisted of 14 Stage I, 40 Stage II, 29 Stage III, and 11 Stage IV patients defined by the Global Initiative for Chronic Obstructive Lung Disease (GOLD) 2014. The mean pack-year cigarette consumption in the COPD group (mean ± SD 66.0 ± 39.9) was greater than that in both the HS (37.1 ± 13.3  *p* < 0.0001 versus COPD) and AR groups (46.5 ± 27.7  *p* < 0.05 versus COPD). As expected, both airflow obstruction and emphysema severity were more severe in the COPD group than in the AR group but there were no significant differences in BMI, serum creatinine levels, frequencies of hypertension, or the use of ICS.

### 3.2. Plasma Concentrations of Cathepsin S and Cystatin C

**Figure 1:**
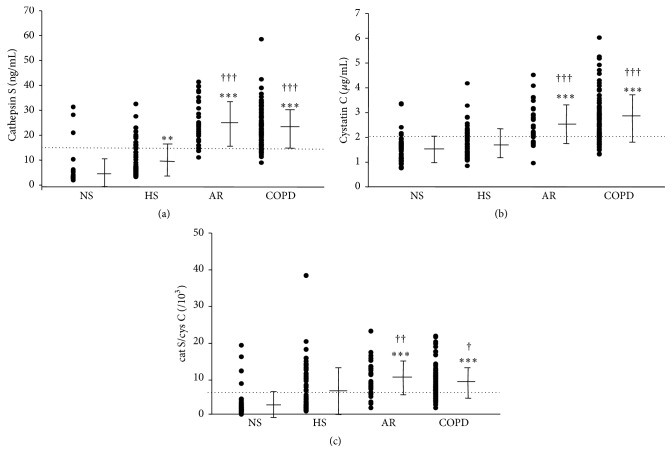
Comparison of plasma levels of cathepsin S (a), cystatin C (b), and cathepsin S/cystatin C (c) between healthy nonsmokers (NS), healthy smokers (HS), subjects at risk (AR), and COPD patients. Distribution of cathepsin S, cystatin C, and cathepsin S/cystatin C among the groups is displayed along with mean ± SD values. Cut points were set at 16.2 ng/mL, 2.1 *μ*g/mL, and 6.4 × 10^−3^, respectively, and displayed as dotted lines, based on the ROC analyses in Figure 2. Mean ± SD is presented. *∗∗* indicates *p* < 0.01; ^*∗∗∗*^ *p* < 0.001 versus NS, ^†^ *p* < 0.05, ^††^ *p* < 0.01, and ^†††^ *p* < 0.001 versus HS.

The mean value and the distribution of plasma concentrations of cathepsin S, cystatin C, and cathepsin S/cystatin C ratios among the four groups are shown in Figure 1. Plasma cathepsin S and cystatin C levels were significantly higher in the AR and COPD groups than those in the NS and HS groups. The cathepsin S/cystatin C ratio was also significantly higher in the AR and COPD groups than in the NS and HS groups. Cathepsin S levels in the HS group were higher than those in the NS group. When we compared plasma cathepsin S and cystatin C levels in GOLD stages I to IV COPD patients, we found no significant differences in the levels of these proteins.

### 3.3. ROC Curves

**Figure 2:**
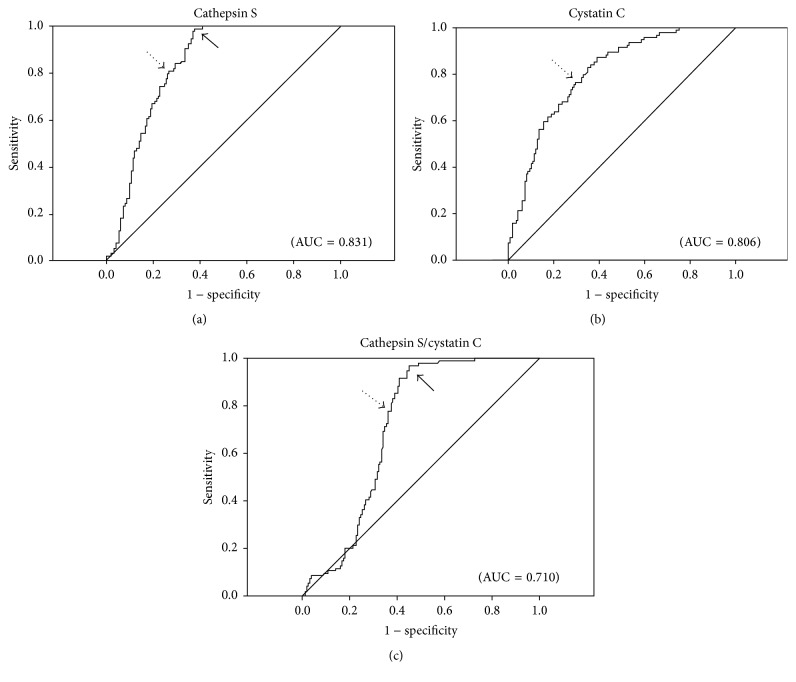
ROC curves for cathepsin S (a), cystatin C (b), and cathepsin S/cystatin C (c). AUC was 0.831, 0.806, and 0.710, respectively. Dotted arrows indicate the best cutpoints for both sensitivity and specificity. Better sensitivities (98% and 97%, resp.) were obtained when the cut points were set at cathepsin S ≥ 12.1 ng/mL and cathepsin S/cystatin C ≥ 4.2 × 10^−3^, indicated by the solid arrows in (a) and (c), and the specificities were 62% and 55%, respectively.

The ROC curves are shown in Figure 2. For plasma cathepsin S, the best threshold for discriminating between patients with versus without COPD was ≥ 16.2 ng/mL (80% sensitivity and 74% specificity). For plasma cystatin C, a threshold of ≥ 2.1 *μ*g/mL had a 77% sensitivity and 71% specificity, whereas for the plasma cathepsin S/cystatin C ratio a value of ≥ 6.4 × 10^−3^ had 78% sensitivity and 64% specificity. Because the ROC curves for plasma cathepsin S and cathepsin S/cystatin C ratios were not symmetrical for sensitivity and specificity (Figure 2), another threshold was determined to improve the sensitivity. A cathepsin S level of ≥ 12.1 ng/mL offered a sensitivity of 98% with 62% specificity. When the plasma cathepsin S/cystatin C ratio was ≥ 4.2 × 10^−3^, the sensitivity was 97% with 55% specificity.

### 3.4. Regression Analyses

**Figure 3:**
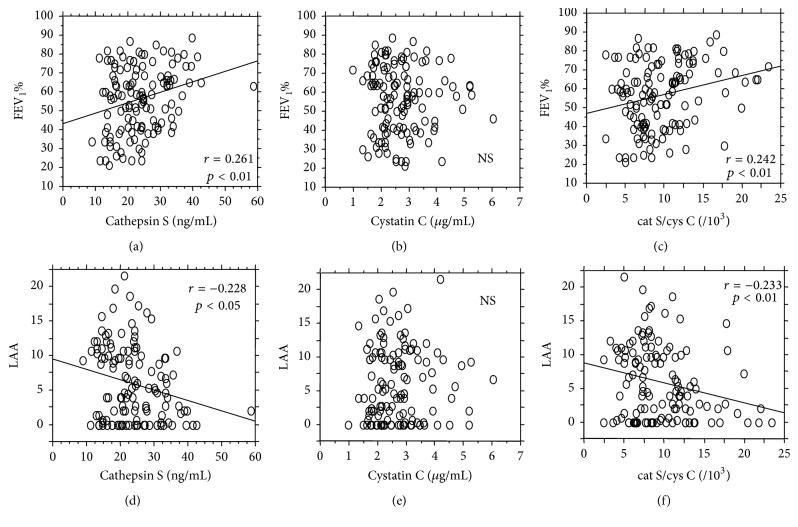
The correlation of cathepsin S, cystatin C, or cathepsin S/cystatin C with either FEV_1_% or LAA score in the AR and COPD groups (*n* = 125). Cathepsin S levels correlated modestly but significantly with FEV_1_/FVC (a) and inversely with the LAA score (d) (*r* = 0.261, *p* < 0.01; *r* = −0.228, *p* < 0.05, resp.); also cathepsin S/cystatin C ratios correlated modestly but significantly with FEV_1_/FVC (c) and inversely with the LAA score (f) (*r* = 0.242, *p* < 0.01; *r* = −0.223, *p* < 0.01, resp.). In contrast, cystatin C levels did not correlate with either FEV_1_/FVC (b) or LAA (e).

We then examined the associations between plasma concentrations of cathepsin S or cystatin C and FEV_1_/FVC or the LAA score in the COPD and AR groups. Unexpectedly, both cathepsin S levels and cathepsin S/cystatin C ratios correlated modestly but significantly with FEV_1_/FVC and inversely with the LAA score, while cystatin C levels did not correlate significantly with either of these parameters (Figure 3).

Table 2 shows that cathepsin S correlated with % FEV_1_ predicted (*p* = 0.045) and %  DL_CO_/*V*_*A*_ predicted (*p* = 0.046). Cystatin C levels correlated positively with age (*p* = 0.01), creatinine levels (*p* = 0.003), %  DL_CO_ predicted (*p* = 0.01), diameter of the abdominal aorta (*p* = 0.005), and male gender (*p* = 0.04). The cathepsin S/cystatin C ratios correlated positively with % FEV_1_ predicted (*p* = 0.04), peak expiratory flow rate (PEF) (*p* = 0.03), *V*_50_ (*p* = 0.04), %  DL_CO_ predicted (*p* = 0.005), and %  DL_CO_/*V*_*A*_ predicted (*p* = 0.01).

Tables 3 and 4 show that after normalizing for the effects of age, BMI, pack-year, creatinine, gender, smoking status, hypertension, and ICS, cathepsin S levels and age were significantly correlated with the development of severe airflow obstruction [*p* = 0.005, odds ratio (OR) = 0.32, and *p* = 0.001, OR = 3.82, resp.]. Also, the cathepsin S/cystatin C ratio (*p* = 0.02, OR = 0.87) and age (*p* = 0.001, OR = 3.02) correlated to severe airway obstruction (Table 3). Serum creatinine levels were also associated with severity of airflow obstruction (Table 3). On the other hand, cathepsin S (*p* = 0.001. OR = 0.28) and the cathepsin S/cystatin C ratio (*p* = 0.002, OR = 0.82) were the strongest factors associated inversely with the presence of severe emphysema (Table 4).

## 4. Discussion

This study shows for the first time that plasma cathepsin S level and cathepsin S/cystatin C ratios are significantly higher in COPD patients compared with healthy smokers and healthy nonsmokers. This suggests that cathepsin S participates in COPD pathogenesis in humans and that plasma cathepsin S and cathepsin S/cystatin C ratios might be potential biomarkers for COPD. It is also intriguing that plasma cathepsin S and cathepsin S/cystatin C ratios tend to be higher in patients with mild airflow limitation and emphysema than those in patients with severe impairment.

Previous studies of animal models of emphysema have implicated cathepsin S in promoting airspace enlargement in mice [9, 19]. Cathepsin S is produced by activated macrophages and dendritic cells. Cathepsin S is also produced by smooth muscle cells and endothelial cells in human atherosclerotic lesions [20] and by adipose tissue [21]. Cathepsin S is a potent elastin-degrading proteinase which retains substantial activity at neutral pH. Thus, cathepsin S could promote airspace enlargement by degrading lung elastin fibers. Cathepsin S is also expressed by antigen-presenting cells such as macrophages and dendritic cells and has crucial activities in processing antigens during MHC class II-mediated immune processes [8, 22]. In this respect, it is noteworthy that a recent hypothesis has suggested that COPD is an autoimmune disease in which anti-elastin, epithelial, and endothelial cell antibodies promote disease progression especially in patients with severe disease [23]. Studies of transgenic mice that overexpress IL-13 and IFN-*γ* in an inducible- and lung-specific manner develop lung inflammation and emphysema due, in part, to increased lung levels of cysteine proteinases including cathepsin S [9, 24]. Cathepsin S contributes to emphysema development in IFN-*γ* overexpressing transgenic mice by promoting epithelial cell apoptosis [19]. Geraghty et al. have suggested a potential contribution of IFN-*γ*-induced cathepsin S expression in macrophages to the development of COPD [7]. However, cathepsin S has not been well studied in humans. Plasma cystatin C levels are increased by renal dysfunction [17] and decreased in patients with aortic aneurysm [18] and COPD patients often have comorbidities including cardiovascular and renal disease, hypertension, and cachexia. Consistent with the protease-antiprotease imbalance hypothesis for COPD, we anticipated that COPD patients would have increased plasma levels of cathepsin S along with reduced plasma levels of its cognate inhibitor, cystatin C [5].

Surprisingly, our data showed that, in addition to plasma cathepsin S levels, plasma levels of cystatin C were also higher in COPD patients compared with the control groups. We show that the differences in plasma levels of cathepsin S and cathepsin S/cystatin C ratios between the COPD patients and the control groups were not due to differences in BMI, and prevalence of cardiovascular disease, as assessed by measuring the diameters of the abdominal aorta and main pulmonary artery, systemic hypertension, or renal dysfunction, as assessed by serum creatinine levels. We also show that age and treatment with ICS, which can influence inflammatory cell activation, were not responsible for the observed differences. The ROC analyses showed that the sensitivity of plasma cathepsin S levels for the diagnosis of COPD was very high (98%) when the threshold was set at ≥ 12.1 ng/mL. The specificity improved from 62% to 80% if the threshold was set at ≥ 16.2 ng/mL but the sensitivity decreased to 74%. These additional analyses suggest that plasma cathepsin S levels may serve as a useful biomarker for COPD. Rokadia et al. also reported that active smokers with COPD had significantly higher plasma levels of cystatin C than healthy controls in accordance with our results [25]. These observations might imply that elevated plasma levels of cystatin C partly reflect the defensive reaction against the effect of increased cathepsin S induced by smoking, still representing an imbalance between the enhanced protease activity and inhibitory effects of cystatin C in patients with COPD.

An unexpected result from our study was that, among the COPD patients and AR subjects, plasma cathepsin S and cathepsin S/cystatin C ratios were inversely correlated with the extent of airflow obstruction and emphysema severity, as assessed by LAA on CT scans, even after normalizing for various confounding factors including age, gender, BMI, renal function, smoking history, the presence of hypertension, and treatment with ICS. Thus, plasma cathepsin S levels, but not cystatin C levels, were significantly increased in patients with AR and with mild COPD when compared with patients with severe airflow limitation and emphysema. One possible explanation for the inverse correlation between plasma cathepsin S and severity of disease among the COPD patients is that cathepsin S is produced in lung tissue including activated macrophages [6], and as COPD severity increases there is progressive destruction of the alveolar walls with loss of source of cathepsin S. Other cellular sources of cathepsin S such as adipose tissue [21] could also be altered quantitatively and qualitatively between the early and late stages of COPD. Chronic airflow limitation assessed by FEV_1_/FVC and % FEV_1_ predicted is positively associated with BMI and malnutrition in a large number of COPD patients [26], and plasma cathepsin S levels are reduced on surgery-related weight loss, linking to reduction of cathepsin S expression in subcutaneous adipose tissue mass [21]. Also, proinflammatory stimuli that differ in expression levels in the lung during the course of COPD differentially regulate cathepsin S mRNA levels and protein secretion by cells [27]. Additionally, the main inhibitor of cathepsin S, cystatin C, inhibits various cysteine proteinases including cathepsins L and K, and lung production of these other cysteine proteinases is also increased in COPD patients [10]. A prior study reported that, unlike COPD patients, patients with asthma have significantly reduced plasma cathepsin S levels when compared to healthy control subjects [28].

Taken together, we speculate that the inverse correlation between plasma cathepsin S and cathepsin S/cystatin C ratios and severity of airflow limitation and emphysema in patients suggestive of COPD that we observed in this study might reflect these complex processes in the pathophysiology of this disease. However, additional cohorts of COPD and asthma will be needed to confirm these findings. Symptomatic smokers without pulmonary function decline are classified as Stage 0 disease and reported to have an increased susceptibility to COPD in GOLD 2001. AR, that is Stage 0 disease, develops COPD in 50% [29]. In addition, productive cough is associated with hastening lung function decline for 5-year follow-up when compared with COPD patients without productive cough [30]. However, there is still little evidence that cough is an important predictor of future progression in Stage 0 patients. Plasma cathepsin S and cathepsin S/cystatin C ratios may be clinically useful biomarkers to discriminate susceptible individuals from nonsusceptible ones and asthmatics prior to development of airflow limitation in COPD. The mechanisms by which cathepsin S may contribute to COPD pathogenesis are beyond the scope of the current study. Although our study did not address any of these potential mechanisms, the observation that these biomarkers are elevated in patients with COPD, especially in early stages, compared with healthy smokers and nonsmokers should help us direct more efforts in this area.

There are several limitations in our study. First, it was impossible to obtain pulmonary function data from subjects in the NS and HS groups. The healthy volunteers (NS and HS) were differentiated from the patients (COPD and AR) since they had no chronic respiratory symptoms leading to visiting hospitals. Second, the mean age of the COPD group was significantly higher than AR and the healthy subjects. However, our results indicate that cathepsin S levels are not affected by age (Table 2). Third, it is not clear which subjects within the AR group will subsequently develop COPD and whether plasma cathepsin S levels and cathepsin S/cystatin C ratios will predict this progression. Longitudinal studies of the AR group would be required to answer these questions. It is also uncertain to what extent the plasma levels of cathepsin S and cystatin C reflect the expression of these proteins in the lung versus other origins such as adipose tissue.

## 5. Conclusion

We have demonstrated significantly elevated plasma levels of cathepsin S and cystatin C, and plasma cathepsin S/cystatin C ratios, in COPD patients versus healthy subjects. Our results suggest that plasma cathepsin S concentrations and cathepsin S/cystatin C ratios may serve as potential biomarkers for COPD.

## Figures and Tables

**Table 1:** Study populations.

	Healthy nonsmoker (NS)	Healthy smoker (HS)	COPD at risk (AR)	COPD
*N*	52	66	31	94
Male/female	50/2	65/1	27/4	89/5
Age	57.9 ± 2.3	56.6 ± 2.9	56.0 ± 5.9	68.3 ± 9.5^*∗∗∗*^
Current/Ex	0/0	40/26	14/17	37/57^#^
Pack-Years	0	37.1 ± 13.3	46.5 ± 27.7	66.0 ± 39.9^†^
BMI	NA	NA	22.4 ± 4.6	21.4 ± 3.1
Serum Cr	NA	NA	0.77 ± 0.16	0.82 ± 0.18
FEV_1_/FVC (%)	NA	NA	78.1 ± 4.2	49.2 ± 13.6^§^
% FEV_1_ predicted	NA	NA	95.5 ± 8.5	55.3 ± 20.9^§^
LAA	NA	NA	1.9 ± 3.1	7.3 ± 5.4^§^
HT (+/−)	0/52	0/66	5/26	17/77
ICS (+/−)	0/52	0/66	1/30	16/78

^*∗∗∗*^ *p* < 0.0001 versus NS, HS, AR; ^#^ *p* < 0.05 versus HS; ^†^ *p* < 0.0001 versus HS, < 0.05 versus AR; ^§^ *p* < 0.0001 versus AR. COPD: chronic obstructive pulmonary disease; BMI: body mass index; Cr: creatinine; FEV_1_/FVC (%): ratio of forced expiratory volume in 1 second to forced vital capacity; LAA: low attenuation area; HT: hypertension; ICS: inhaled corticosteroid.

**Table 2:** Simple regression analysis.

	Cathepsin S		Cystatin C		Cat S/Cys C (/10^3^)	
	*R*	*p*	*R*	*p*	*R*	*p*
Age	0.033	0.72	0.226	0.01^*∗*^	−0.128	0.16
Pack-Year	0.099	0.27	0.096	0.29	−0.001	0.99
BMI	0.175	0.052	−0.042	0.65	0.132	0.14
Serum Cr	0.164	0.07	0.266	0.003^*∗∗*^	−0.093	0.30
% VC	−0.0004	0.997	−0.054	0.55	0.076	0.40
FEV_1_/FVC	0.261	0.003^*∗∗*^	−0.014	0.88	0.242	0.007^*∗∗*^
% FEV_1_ predicted	0.179	0.045^*∗*^	−0.020	0.83	0.188	0.04^*∗*^
PEF	0.083	0.36	−0.1664	0.07	0.199	0.03^*∗*^
*V*_50_	0.131	0.15	−0.110	0.23	0.187	0.04^*∗*^
% DL_CO_ predicted	0.089	0.37	−0.251	0.01^*∗*^	0.277	0.005^*∗∗*^
% DL_CO_/*V*_*A*_	0.197	0.046^*∗*^	−0.120	0.23	0.244	0.01^*∗*^
LAA	−0.228	0.01^*∗*^	0.033	0.71	−0.233	0.009^*∗∗*^
mPA	0.112	0.24	0.139	0.14	−0.054	0.58
abAo	0.181	0.06	0.267	0.005^*∗∗*^	0.045	0.64
Current/Ex (51/74)	24.8/22.8	0.17	2.78/2.65	0.44	9.77/9.42	0.65
HT (+/−) (22/103)	24.5/23.4	0.57	2.74/2.70	0.57	9.68/9.54	0.85
ICS (+/−) (17/108)	20.1/24.2	0.06	2.59/2.72	0.59	8.36/9.75	0.21
M/F (116/9)	23.7/23.4	0.93	2.66/3.32	0.04^*∗*^	9.69/7.90	0.22

^*∗*^ *p* < 0.05; ^*∗∗*^ *p* < 0.01. Cat: cathepsin; Cys: cystatin; BMI: body mass index; Cr: creatinine; VC: vital capacity; FEV_1_/FVC: ratio of forced expiratory volume in 1 second to forced vital capacity; FEV_1_: forced expiratory volume in 1 second; PEF: peak expiratory flow rate; *V*_50_: the air flow rate at 50% vital capacity; DL_CO_: diffusing capacity of the lung for carbon monoxide; DL_CO_/*V*_*A*_: diffusing capacity of the lung for carbon monoxide divided by the alveolar volume; LAA: low attenuation area; mPA: main pulmonary artery; abAo: abdominal aorta; Ex: ex-smoker; HT: hypertension; ICS: inhaled corticosteroid; M: male; F: female.

**Table 3:** Logistic regression analysis for severe airflow limitation (% FEV_1_ predicted < 50).

	Cathepsin S and % FEV_1_predicted < 50				Cat S/Cys C (/10^3^) and % FEV_1_ predicted < 50			
	*χ* ^2^	*p*	OR	95% CI	*χ* ^2^	*p*	OR	95% CI
Cat S (10 increments)	7.82	0.005^*∗∗*^	0.32	0.14–0.71	—	—	—	—
Cat S/Cys C (/10^3^)	—	—	—	—	5.19	0.02^*∗*^	0.87	0.77–0.98
Age (10 increments)	12.14	0.001^*∗∗*^	3.82	1.80–8.12	10.76	0.001^*∗∗*^	3.02	1.56–5.84
BMI	2.28	0.13	0.90	0.79–1.03	2.77	0.10	0.89	0.77–1.02
PY (10 increments)	2.46	0.12	1.11	0.97–1.27	1.21	0.27	1.07	0.95–1.22
Serum Cr	5.05	0.02^*∗*^	0.02	0.001–0.60	6.35	0.01^*∗*^	0.014	0.0005–0.39
Male	3.35	0.07	11.93	0.84–169.5	4.10	0.04^*∗*^	15.13	1.09–209.5
Ex-smoker	1.44	0.23	1.88	0.67–5.28	2.97	0.09	2.45	0.88–6.79
HT (−)	2.18	0.14	0.40	0.12–1.35	2.09	0.15	0.42	0.13–1.36
ICS (−)	0.001	0.97	1.03	0.26–4.07	0.044	0.83	0.87	0.23–3.23

^*∗*^ *p* < 0.05; ^*∗∗*^ *p* < 0.01. FEV_1_: forced expiratory volume in 1 second; Cat: cathepsin; Cys: cystatin; OR: odds ratio; CI: confidence interval; BMI: body mass index; PY: pack-years; Cr: creatinine; HT: hypertension; ICS: inhaled corticosteroid.

**Table 4:** Logistic regression analysis for significant emphysematous change (LAA ≥ 8.0).

	Cathepsin S and LAA ≥ 8.0				Cat S/Cys C (/10^3^) and LAA ≥ 8.0			
	*χ* ^2^	*p*	OR	95% CI	*χ* ^2^	*p*	OR	95% CI
Cat S (10 increments)	12.21	0.001^*∗∗*^	0.28	0.14–0.57	—	—	—	—
Cat S/Cys C (/10^3^)	—	—	—	—	9.83	0.002^*∗∗*^	0.82	0.72–0.93
Age (10 increments)	5.72	0.02^*∗*^	2.08	1.14–3.78	3.76	0.052	1.72	0.99–2.98
BMI	3.45	0.06	0.87	0.75–1.01	4.36	0.04^*∗*^	0.85	0.73–0.99
PY (10 increments)	1.92	0.17	1.09	0.96–1.24	0.74	0.39	1.05	0.94–1.19
Serum Cr	0.37	0.54	0.40	0.02–7.71	1.55	0.21	0.16	0.009–2.88
Male	1.28	0.26	3.17	0.43–23.4	2.51	0.11	4.71	0.69–32.1
Ex-smoker	0.31	0.58	1.30	0.51–3.29	1.46	0.23	1.76	0.70–4.42
HT (−)	0.84	0.36	0.58	0.18–1.86	0.77	0.38	0.60	0.20–1.87
ICS (−)	0.07	0.79	0.85	0.25–2.89	0.21	0.65	0.76	0.23–2.51

^*∗*^ *p* < 0.05; ^*∗∗*^ *p* < 0.01. LAA: low attenuation area; Cat: cathepsin; Cys: cystatin; OR: odds ratio; CI: confidence interval; BMI: body mass index; PY: pack-years; Cr: creatinine; HT: hypertension; ICS: inhaled corticosteroid.

## Abbreviations

abAo: Abdominal aorta
AR: At risk
BMI: Body mass index
Cat: Cathepsin
CI: Confidence interval
COPD: Chronic obstructive pulmonary disease
Cr: Creatinine
Cys: Cystatin
DL_CO_: Diffusing capacity of the lung for carbon monoxide
DL_CO_/*V*_*A*_: Diffusing capacity of the lung for carbon monoxide divided by the alveolar volume
ELISA: Enzyme-linked immunosorbent assay
Ex: Ex-smoker
F: Female
FEV_1_: Forced expiratory volume in 1 second
FEV_1_/FVC: Ratio of forced expiratory volume in 1 second to forced vital capacity
FVC: Forced vital capacity
GOLD: Global initiative for chronic obstructive lung disease
HS: Healthy smoker
HT: Hypertension
ICS: Inhaled corticosteroid
LAA: Low attenuation area
M: Male
mPA: Main pulmonary artery
NS: Nonsmoker
OR: Odds ratio
PEF: Peak expiratory flow rate
PFT: Pulmonary function tests
PY: Pack-years
ROC: Receiver operating characteristic
VC: Vital capacity
*V*_50_: The air flow at the 50% vital capacity.
